# *In vitro* dynamic pharmacokinetic/pharmacodynamic(PK/PD) modeling and PK/PD cutoff of cefquinome against *Haemophilus parasuis*

**DOI:** 10.1186/s12917-015-0343-7

**Published:** 2015-02-13

**Authors:** Xia Xiao, Jian Sun, Yi Chen, Rui-Juan Huang, Ting Huang, Guilin Gary Qiao, Yu-Feng Zhou, Ya-Hong Liu

**Affiliations:** College of Veterinary Medicine, National Reference Laboratory of Veterinary Drug Residues (SCAU), South China Agricultural University, Guangzhou, 510642 China; 8725, John J Kingman Rd, MS 6201, Ft Belvoir, VA 22060-6201 USA; Jiangsu Co-Innovation Centre for Prevention and Control of Important Animal Infectious Diseases and Zoonoses, Yangzhou, Jiangsu People’s Republic of China

**Keywords:** Pharmacokinetic/pharmacodynamic, Cefquinome, PK/PD cutoff, Monte Carlo simulation

## Abstract

**Background:**

*Haemophilus parasuis* (*H. parasuis*) causes Glässer’s disease and multisystem infectious disease. It is one of the major causes of nursery mortality in swine herds. Cefquinome (CEQ) is proposed for the treatment of pigs against respiratory tract infection. However, few studies have investigated the PK/PD characteristics and PK/PD cutoff of this drug against *H. parasuis*.

**Results:**

A total of 213 *H. parasuis* strains were isolated from diseased pigs in China. The minimal inhibitory concentrations (MICs) of CEQ against these isolates were determined. The MIC_50_ and MIC_90_ values were 0.125 and 8 mg/L, respectively. An *in vitro* dynamic PK/PD infection model was used to investigate the antimicrobial effect of CEQ against *H. parasuis* strain of serotype 5. The target values of CEQ for 3-log_10_-unit and 4-log_10_-unit decreases effects were the percent time that CEQ concentrations were above the minimum inhibitory concentration (T% > MIC) of 61 and 71 respectively. According to Monte Carlo simulation, the PK/PD cutoff for CEQ against *H. parasuis* was 0.06 mg/L. The suggested dose regimen was 4 mg/kg/12 h BW.

**Conclusions:**

The value of PK/PD surrogate marker T% > MIC is of great utility in CEQ clinical usage. The very first CEQ PK/PD cutoff provide fundamental data for CEQ breakpoint determination. A more desirable dose regimen against *H. parasuis* was provided for CEQ using in China district.

## Background

*H. parasuis* is a commensal bacterium of the upper respiratory tract of swine [1]. Under certain conditions, *H. parasuis* can invade the body and cause Glässer’s disease as well as other severe diseases, such as arthritis, fibrinous polyserositis and meningitis [2]. Moreover, *H. parasuis* can invade into the AOC-45 porcine aorta endothelial cells [3] and its endotoxin is able to induce disseminated intravascular coagulation, which results in the formation of microthrombi in some important organs [2]. In addition, *H. parasuis* frequently interacts with porcine reproductive respiratory syndrome virus (PRRSv) and causes more economic losses [4,5]. *H. parasuis* has numerous serovars, some of which show severe virulence and cause death within 4 days such as serovar 1, 5, 10, 12, 13 and 14 [6]. Of them, serovar 5 and 4 are the most prevalent types isolated in China [7], Danmark [8], Germany [6], the USA [9], Japan [10] and Spain [11], whereas serovars 5 and 13 are prevalent in Australia [12]. Due to the serovar diversity and lack of cross-reaction among the serovars, it is hard to develop an effective cross-protective vaccine [13]. Therefore pharmacotherapy still plays a significant role in treating *H. parasuis* infections.

Cefquinome (CEQ) is a fourth generation cephalosporin antibiotic that is solely developed for veterinary use. It possesses high stability to β-lactamases and is active against Gram-positive and Gram-negative bacteria. The European Medicines Agency (EMA) proposes CEQ for the treatment of pigs against respiratory tract infections with a dosage regimen of 2 mg/kg/24 h bodyweight (BW) for three to five days [14]. As one of the major pathogens of respiratory tract of pigs, few studies have investigated the killing pattern and PK/PD characteristics of this drug against *H. parasuis*, moreover, the Clinical and Laboratory Standards Institute (CLSI) subcommittee on Veterinary Antimicrobial Susceptibility Testing (VAST) which establishes veterinary clinically breakpoints [15] has not established breakpoints for *H. parasuis*. The value of PK/PD surrogate which is of great utility in CEQ clinical usage and the CEQ PK/PD cutoff providing fundamental data for CEQ breakpoint determination should be illuminated.

In this study, the minimal inhibitory concentrations (MICs) of CEQ against 213 *H. parasuis* isolates identified in China were determined. An *in vitro* PK/PD infection model has been used to investigate the antimicrobial effect of CEQ against *H. parasuis* strain of serotype 5, which is highly virulent and one of the most prevalent serotypes in China [7]. A 5,000-subjects Monte Carlo simulation has been done to derive a PK/PD cutoff based on three aspects: MIC distributions of CEQ against *H. parasuis*, pharmacokinetic/pharmacodynamic (PK/PD) indices and Pharmacokinetics of CEQ in swine obtained in a previous study [16]. A more rational regimen was recommended according to Monte Carlo simulation results.

## Methods

### Animal ethics

All husbandry practices and experimental operations were performed with full consideration of animal welfare. Research ethical approval was granted by the South China Agriculture University Animal ethics committee (2014-03).

### Strains and antibiotic

*H. parasuis* strain representing serovar 5 was kindly provided by Professor Ming Liao, College of Veterinary Medicine, South China Agricultural University, Guangzhou, Guangdong Province, China. It was CEQ sensitive to MIC of 0.003 mg/L. The other 213 *H. parasuis* isolates used in this study were derived from diseased pigs suffering fibrinous polyserositis, meningitis and polyarthritis diagnosed in China during August 2010 to July 2011. Serotypes of these isolates are not known. All the strains were stored at −80°C in milk. Prior to use, they were streaked on a tryptone soya agar (TSA) (Oxoid Ltd., Basingstoke, Hampshire, UK) added with 5% new-born calf serum (Guangzhou Ruite Bio-tec Co., Ltd., Guangdong, China) and 2% beta-Nicotinamide adenine dinucleotide trihydrate (NAD) (Qingdao Hope Bio-Technology Co., Ltd., Shandong, China). Tryptone soya broth (Oxoid Ltd., Basingstoke, Hampshire, UK) added with the same amount of NAD and new-born calf serum was used to culture *H. parasuis*. CEQ sulfate injection (25 mg/mL) was purchased from Hebei Yuanzheng Pharmaceutical Company (Hebei, China).

### MICs

Given the unavailability of a CLSI approval method for *H. parasuis*, MICs were conducted in accordance with the CLSI VET01-A4 recommendations for *Actinobacillus pleuropneumoniae* [17]. The quality control strain was *Actinobacillus pleuropneumoniae* ATCC 27090. The values of MIC_50_ and MIC_90_, inhibiting the growth of at least 50% and 90% of isolates in a test population, respectively, were calculated in this study following the methods previously published [18].

### ***In vitro*** dynamic PK/PD modeling

The *in vitro* one-compartment PK/PD infection model was constructed according to previously described method [19] with some improvements. The model system contained fresh trypticase soy broth reservoir, central compartment and waste storage compartment. These compartments were connected with silicone tubes. The broth was pumped from the reservoir to the central compartment through a peristaltic pump, which is controlled digitally. An inverted 15-mL centrifuge tube with a cellulose ester membrane (0.2-μm pore size) covering the top was placed in the central compartment to prevent bacteria from flowing out to the medium. Below the membrane, a magnetic stir bar was placed on the bottom of the central compartment. The stir bar mixed the broth and enabled the drug to fully contact with the bacteria. The temperature of the reservoir and central compartment was controlled between 36 and 37°C via water bath. The entire experimental system was placed in a UV-sterilized worktable. The reservoir and central compartment contained 700 and 60 mL of trypticase soy broth, respectively. Fresh broth pumped into the central compartment, and the same volume culture media pumped into the waste container. To simulate intravenous injection pharamcokinetics of CEQ in swine with this equipment, the flow rate was 0.37 mL/min. The drug was administered into the central compartment via the sampling port at zero time point.

### ***In vitro*** time kill curves of CEQ

A 12 h culture of bacteria with optical density (OD) value of approximately 0.09 was added to the central compartment [10^7^ colony forming unit (cfu)/mL] and incubated at 35°C to 37°C for 30 min to condition the bacteria to the new environment. Different doses of CEQ (0.28, 0.68, 4.03, 9.79, 15.26, 37.08, 90.13, 140.50, 219.03, 341.45 μg) or control (sterile normal saline) were introduced into the central compartment, and the peristaltic pump was turned on immediately. A 150 μL aliquot was obtained for bacterial counting at time points of 0, 3, 6, 9, 12 and 24 h. Samples were diluted properly with sterile normal saline, and 25-μL of aliquots of the last four diluted samples were plated onto the TSA plates and incubated at 37°C for 24 h. To monitor drug concentrations, 200-μL aliquots were also obtained at 1, 6, 12 and 24 h and centrifuged at 8,000 rpm at 4°C for 10 min. The supernatant was stored at −80°C and analyzed within 1 month. All experiments were performed in duplicate on different days.

### Pharmacokinetics and PK/PD analysis

The samples were analyzed for CEQ concentration using the method reported previously [19]. The PK data were analyzed using Phoenix WinNonlin 6.0 software (Pharsight Co. Ltd.). A T% > MIC value during a 24-h interval was calculated using the pharmacokinetic and MIC data for each time kill curve. The *in vitro* drug effect was quantified by changes in log_10_ cfu counts between 24 and 0 h. Data were analyzed using sigmoid *E*_max_ model WINNONLIN software (version 6.1; Pharsight, CA, USA) per the following equation:

$$ E={E}_0+\frac{E_{\max}\times {C}_e^N}{E{C}_{50}^N+{C}_e^N} $$

where *E*_0_ is the change in log_10_ cfu/mL after 24-h incubation in the control sample compared to the initial inoculum. *E*_*max*_ is the difference in effect between the greatest amount of growth (as seen for the growth control, E_0_) and the greatest amount of kill. *C*_e_ is the T% > MIC in the effect compartment. EC_50_ is the T% > MIC value producing a 50% reduction in bacterial counts from the initial inoculum, and *N* is the Hill coefficient that describes the steepness of the T% > MIC–effect curve. Three levels of growth inhibition were calculated. T% > MIC for bacteriostatic and bactericidal actions are values that produce *E* = 0 (no change in bacterial counts after 24 h incubation) and *E* = − 3 (a 3 log10 or 99.9% reduction of the original inoculum counts after 24 h incubation), respectively. Bacterial eradication is the lowest T% > MIC that provides a 4 log_10_ reduction.

### Monte Carlo simulation

Based on a previous pharmacokinetic study of CEQ in pigs [19], MIC distribution, and the value of PK/PD target indices obtained in this study, a 5,000-subjects Monte Carlo simulation was conducted using Crystal Ball Professional V7.2.2 software. The time above MIC was calculated using the following equation:

$$ \mathrm{C}=\frac{{\mathrm{k}}_{\mathrm{a}}\mathrm{F}{\mathrm{X}}_0}{{\mathrm{k}}_{\mathrm{a}}\hbox{-} \mathrm{k}}\left({\mathrm{e}}^{\hbox{-} \mathrm{k}\mathrm{t}}\hbox{-} {\mathrm{e}}^{\hbox{-} {\mathrm{K}}_{\mathrm{a}}\mathrm{t}}\right) $$

Where C is the value of MIC, k_a_ is the absorption half life, F is the bioavailability, X_0_ is the dose of antibiotic and k is the elimination half life.

All the PK parameters were assumed to be normally distributed in the form of mean values and confidence intervals (Table 1). MICs were fixed at single values from 0.0015 mg/L to 16 mg/L. The PK/PD cutoff is the MIC, at which the probability of target attainment (PTA) for 3-log_10_-unit decrease equals 90% under clinical recommended dose. Scenarios were simulated separately for the IM administration, which were single-dose administrations of 2, 4, 8, 16, 32 and 60 mg/kg BW and doses of 1, 2, 4, 6, 8 and 16 mg/kg BW administered in two equal doses at 12-h interval.

**Table 1:** **Serum pharmacokinetic parameters after IV administration at 2 mg/kg bodyweight in a two-compartment open model used for Monte Carlo simulation**

**Parameter (units)**	**Mean value**	**SD**
T_1/2ka_ (h)	0.06	0.03
T_1/2β_ (h)	2.34	0.09
CL/F(L/kg)	0.09	0.03
F(%)	116.29	14.72

## Results

### MIC distribution

**Figure 1:**
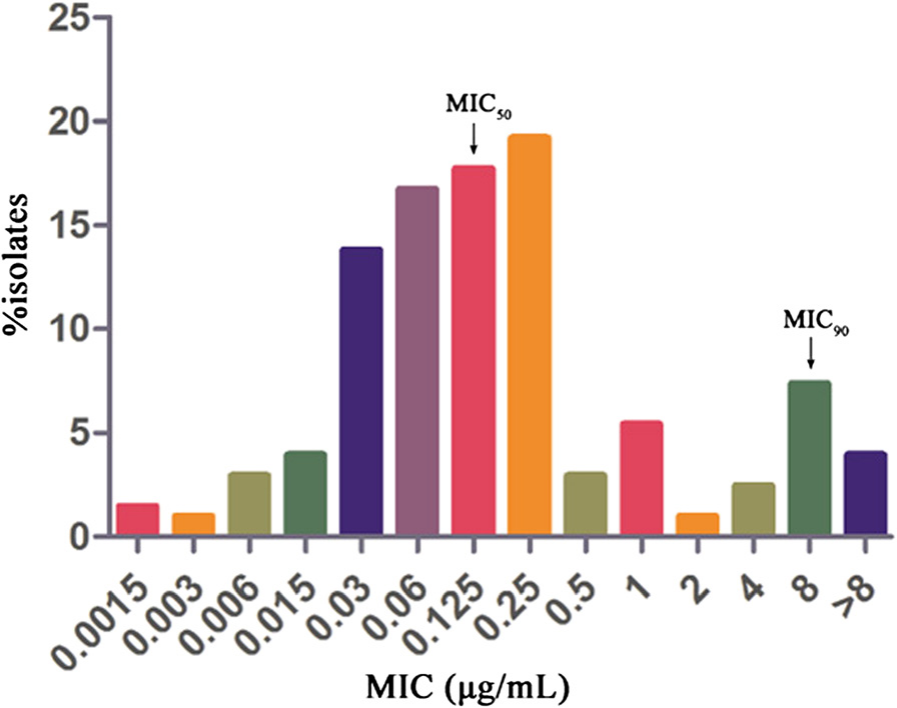
**Minimum inhibitory concentrations (MIC) of cefquinome against** ***H. parasuis.*** (213 strains in total).

MICs of CEQ against 213 *H. parasuis* isolates were diverse, ranging from 0.0015 mg/L to 16 mg/L. The percentages for each MIC (0.0015, 0.003, 0.006, 0.015, 0.03, 0.06, 0.125, 0.25, 0.5, 1, 2, 4, 8 and 16 mg/L) were 1.48%, 0.96%, 2.95%, 3.94%, 13.79%, 16.75%, 17.73%, 19.21%, 2.96%, 5.42%, 0.99%, 2.46%, 7.39% and 3.94%, respectively (Figure 1). Although a low peak of MIC was observed at 8 mg/L, the MICs distributed in a normal distribution pattern basically with a definite peak at 0.25 mg/L. The MIC_50_ and MIC_90_ were determined to be 0.125 and 8 mg/L, respectively.

### ***In vitro*** dynamic PK/PD modeling

**Figure 2:**
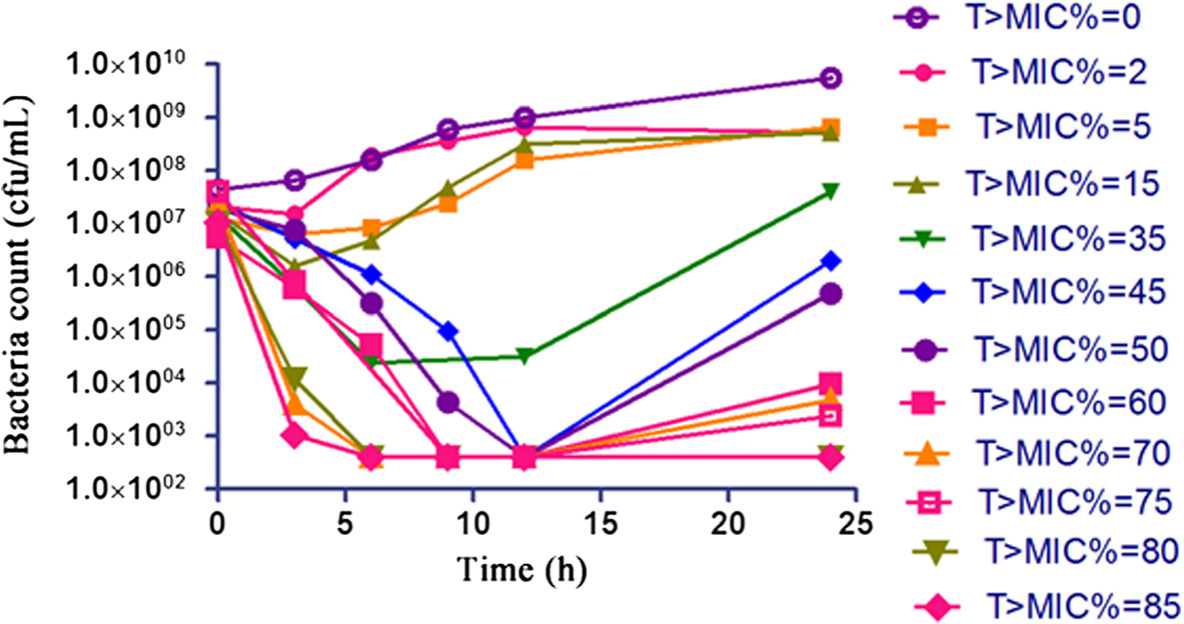
**Time–kill curve of cefquinome against** ***H. parasuis*** **in** ***in vitro*** **PK/PD model.**

**Figure 3:**
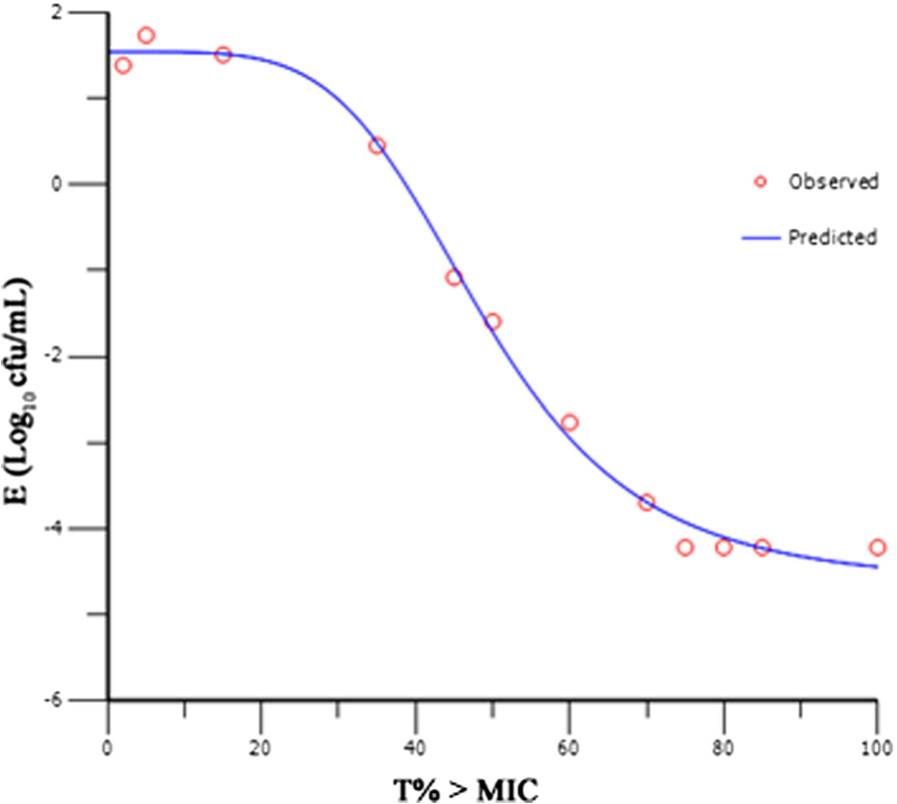
**Sigmoid** ***E***_max_ **model relationships between antimycoplasmal effect [E, log**_1_ **(cfu/mL)] and T% > MIC of cefquinome in** ***in vitro*** **PK/PD model against** ***H. parasuis*** **with an inoculum size of 1 × 10** ^**7**^ **cfu/mL.**

The pharmacokinetics of CEQ in pigs was well simulated by this *in vitro* model with a relative deviation below 6%. For bacterial counting, the limit of determination was 400 cfu/mL. The CEQ inhibited *H. parasuis* moderately when T% > MIC values were equal to or below 15 within 24 h (Figure 2). When T% > MIC value were 35 and 50, CEQ gained a 3-log_10_-unit and a 4-log_10_-unit decrease, respectively, at 9 h to 12 h, but regrowth was observed at 24 h. When T% > MIC increased to the values of 60 and 70, CEQ completely killed *H. parasuis* without regrowth in 24 h (3-log-unit and 4-log-unit decrease, respectively). CEQ is considered to be a time-dependent drug. The *in vitro* antimicrobial effect of CEQ was described successfully using sigmoid *E*_max_ model by the suitable PK/PD surrogate marker T% > MIC (Figure 3). The estimated *E*_0_, EC_50_ and *E*_max_ are listed in Table 2. The target values of 3-log_10_-unit and 4-log_10_-unit decreases for T% > MIC were 61 and 71, respectively.

**Table 2:** **Pharmacodynamic analysis of data acquired from** ***in vitro*** **time-killing studies for cefquinome against** ***H. parasuis***

**Parameter (units)**	**Value**
Log *E*_max_ (cfu/mL)	−4.7
Log *E*_0_ (cfu/mL)	1.6
EC_50_ (h)	48.9
T% > MIC (bacteriostatic) (h)	38.7
T% > MIC (bactericidal) (h)	60.9
T% > MIC (bacterial elimination) (h)	70.9
Slope (N)	4.7

Where *E*_0_ is the change in log_10_ cfu/mL after 24 h incubation in the control sample compared to the initial inoculum. *E*_max_ is the difference in effect between the greatest amount of growth (as seen for the growth control, *E*_0_) and the greatest amount of kill. *C*_e_ is the T% > MIC in the effect compartment. *EC*_50_ is the T% > MIC value producing a 50% reduction in bacterial counts from the initial inoculum, and *N* is the Hill coefficient that describes the steepness of the T% > MIC–effect curve.

### Monte Carlo simulation

**Figure 4:**
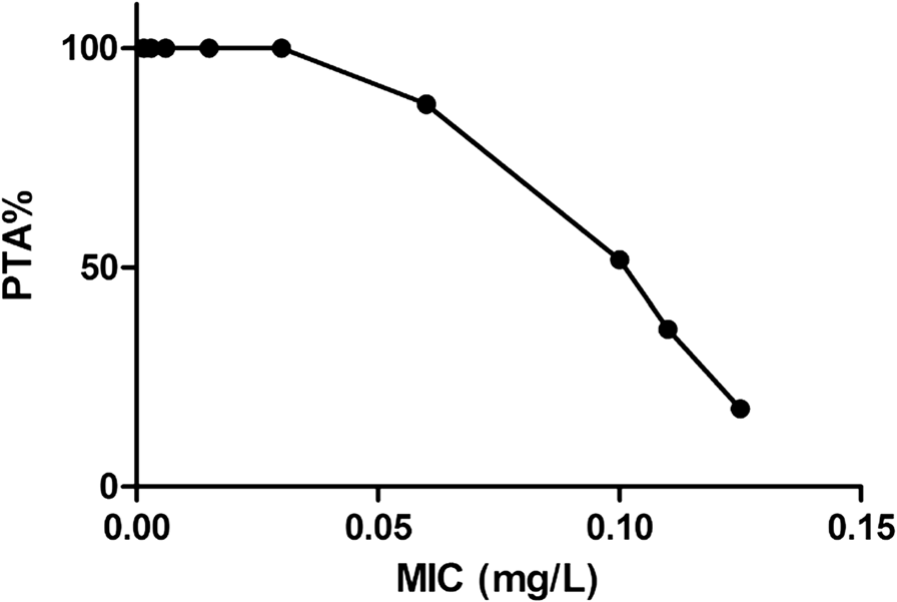
**Probability of target attainment (PTA) for the treatment with cefquinome dose of 2 mg/kg/24 h.**

**Figure 5:**
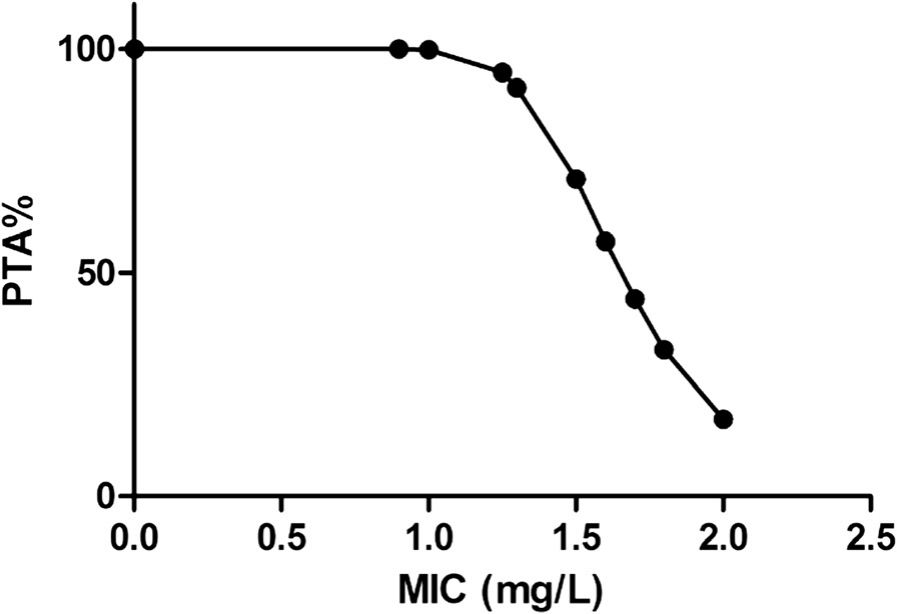
**Probability of target attainment (PTA) for the treatment with cefquinome dose of 4 mg/kg/12 h.**

The PTAs following CEQ administration at dose of 2 mg/kg/24 h was shown in Figure 4. For the recommended 2 mg/kg/24 h BW dose administered by IM, PTA > 90% could only be achieved for MIC < 0.06 mg/L. That is to say, the PK/PD cutoff for CEQ against *H. parasuis* was 0.06 mg/L. The PTAs for each effect and the PK/PD cutoffs with different drug regimens are listed in Table 3. A recommended single IM administration of CEQ dose (2 mg/kg/24 h BW) could not achieve PTA > 90%, neither did an increased dose of 60 mg/kg/24 h BW for all the strains used in this study. However, a PTA of 90.03% was acquired by splitting the daily dose of (32 mg/kg/24 h BW) into two equal doses given at a 12-h interval (16 mg/kg/12 h). In consideration of the PTA (85.79%) following CEQ administration at dose of 4 mg/kg/12 h, which was close to 90%, the recommended dose regimen was defined as 4 mg/kg/12 h. The PTAs following CEQ administration at dose of 4 mg/kg/12 h was shown in Figure 5.

**Table 3:** **Probability of target attainment (PTA) and breakpoints of cefquinome against** ***H. parasuis*** **with recommended regimens (2 mg/kg/24 h and 4 mg/kg/12 h)**

**Drug regimens**	**PTA for T% > MIC = 61**	**PTA for T% > MIC = 71**	**Susceptibility breakpoint (mg/L)**
1 mg/kg/12 h	76.87	72.52	0.3
2 mg/kg/12 h	82.19	80.63	0.6
4 mg/kg/12 h	85.79	84.37	1.3
6 mg/kg/12 h	86.46	85.88	1.9
8 mg/kg/12 h	87.64	86.76	2.6
16 mg/kg/12 h	90.03	88.55	5.2
2 mg/kg/24 h	44.79	28.18	0.06
4 mg/kg/24 h	61.77	43.52	0.15
8 mg/kg/24 h	76.91	61.6	0.3
16 mg/kg/24 h	81.25	77.21	0.6
32 mg/kg/24 h	86.06	82.21	1.2
60 mg/kg/24 h	86.64	84.74	2.2

## Discussion

*H. parasuis* frequently causes Glässer’s disease, which is often treated with sulfanilamide, quinolones and cephalosporins. However, with these drugs wildly used, the resistant *H. parasuis* isolates are emerging quickly [20]. The most important factor in resistance emergence and spread is drug exposure, especially, the exposure to sub- therapeutic drug concentrations [21]. The PK/PD modeling determines the exposure-activity relationships. Using the regimens based on appropriate PK/PD targets will prevent the emergence of resistance [22]. So it is of great importance to administer antibiotics to animals using regimens that will attain appropriate PK/PD targets [23]. In this PK/PD analysis, T% > MIC which was considered as the PK/PD index of most cephalosporins [24] correlated well with *in vitro* drug efficiency. Through the Sigmoid *E*_max_ modeling, the PK/PD target for bactericidal effect and elimination action was T% > MIC = 61 and 71, respectively*.* The results reported here were highly consistent with the published data that the greatest efficacy of cephalosporins in several animal infection models could be achieved as the value of T% > MIC reached 60% - 70% of the dosing interval against *Streptococci* or *Enterobacteriaceae* [25].

PK/PD cutoffs are important tools to set susceptibility breakpoints, which have also been used by regulatory agencies, such as EUCAST and VAST, to refine the susceptibility breakpoint [26]. Monte Carlo simulation provides great advantage using drug exposure–effect relationship [27] which considers pharmacokinetic variation in target animals, MIC distribution and PK/PD indices in defining the PK/PD cutoffs. The PK/PD cutoff of CEQ against *H. parasuis* was 0.06 mg/L under EMA recommended dosage (2 mg/kg/day). This PK/PD cutoff value was similar with the clinical breakpoint values of cefotaxime, a third generation cephalosporin antibiotic, against *Pasteurella multocida* (0.03 mg/L) or *Neisseria meningitides* (0.12 mg/L). Similarities were also found in values of clinical breakpoints of cefotaxime (0.12 mg/L), ceftriaxone (0.12 mg/L), cefixime (0.12 mg/L), cefpodoxime (0.25 mg/L) and ceftaroline (0.03 mg/L) against *Haemophilus influenzae,* another bacteria of *Haemophilus spp.* in human clinic (EUCAST, 2014) [28]*.* However, this breakpoint was much lower than the PK/PD cutoff of cefotaxime (1 mg/L), ceftriaxone (1 mg/L), ceftazidime (4 mg/L) and cefepime (4 mg/L) (EUCAST, 2014) [28]. As the half-life and the PK/PD targets of CEQ are almost identical with those of the cephalosporins (1.5- 2.5 h) [25,29], the main reason for the difference may result from the dosing interval. PK/PD cutoff values for those drugs were based on drugs administered two or three times one day except ceftriaxone while single dose administration as recommended by EMA was chose in this study for CEQ. Compared to single dosing, the concentrations of these drugs after administration with multiple doses within 24 h could maintain a relatively high level, resulting in a higher MIC value of threshold for attaining the same T% > MIC value. Though the PK/PD cutoff had been determined, to establish a susceptibility breakpoint of CEQ, the clinical data are needed.

Under recommended dose regimen of 2 mg/kg/24 h, the PTAs for reach the T% > MIC of 61 and 71 were 44.8% and 28.2%. This seems to indicate that only less than half of the infections could be cured and less than a third of infections could be bacterial eradicated*.* In order to improve clinical response to therapy and reduce selection pressure for antimicrobial resistance during therapy, increasing doses were needed. With the increasing of the total amount of drug, the PTA increased accordingly in both single doses and 2 splitting dose regimens. However, the same daily amount of CEQ administered once-daily or twice-daily, such as 1 mg/kg/12 h and 2 mg/kg/24 h, resulted in different PTAs (76.87% and 44.79%, respectively) implying that splitting dose would reach higher PTA than a single dose administration. Moreover, compared to the dose splitting scenario, the PTA increased much less under single dosing, although the total amount of drug was increased. Therefore, dosing interval change, such as daily dose splitting, offers a much more attractive strategy in improving the clinical curing rate. Similar conclusion was conducted in cefprozil against *H. influenzae* that once daily dosing of cefprozil provided less activity compared with twice daily [30].

A theoretical regimen (i.e., 16 mg/kg/12 h) was recommended based on the PTA for T% > MIC of 61 over 90%. However, considering the economic impact and the therapeutic outcome, 4 mg/kg/12 h is suggested as a more suitable dose regimen, which is less expensive for farmers, but its PTA (85.79%) for T% > MIC of 61 is closer to 90%. Additionally, a much less drug amount dosage can be more eco-friendly, cost-effective and minimize the drug residue burden on public health via human food consumption.

Under the EMA recommended regimen, because of the single dose and short elimination half-life of CEQ*,* the derived PK/PD cutoff was 0.06 mg/L. It was lower than MIC_50_ (0.125 mg/L) of isolates from China. This result indicates that no more than half of the infection in China district could use CEQ for curing under recommend regimen. While, for the dose of 4 mg/kg/12 h recommended in this study, the PK/PD cutoff was 1.3 mg/L. Though it didn’t cover all the MIC value, it covered more than 85% of the MIC distribution. Howerver, one point should be paid attention to is that the strains used in this simulation were limited in china. It cannot stand for the MIC distribution of the world. For international CEQ dose regimen against *H. parasuis*, more MICs of *H. parasuis* strains in a worldwide scale should be taken into account. Although further clinical studies are needed to confirm the modelling results, the present study could provide fundamental data for CEQ susceptibility test and improve the use of CEQ for swine health to a certain extent.

## Conclusions

In conclusion, this study established an *in vitro* dynamic PK/PD modelling of CEQ against *H. parasuis*. The target values of CEQ for 3-log_10_-unit and 4-log_10_-unit decreases effects were T% > MIC of 61 and 71 respectively. The very first CEQ PK/PD cutoff (0.06 mg/L) which is of great utility in CEQ susceptibility breakpoint determination and dosing design were derived based on Monte Carlo simulation. A more desirable dose regimen against *H. parasuis* was determined to be 4 mg/kg/12 h in China.

## Abbreviations

T% > MIC: The percent time that CEQ concentrations were above the minimum inhibitory concentration
*H. parasuis*: *Haemophilus parasuis*
CEQ: Cefquinome
PK/PD: Pharmacokinetic/pharmacodynamic
MIC: Minimal inhibitory concentrations
PRRSv: Porcine reproductive respiratory syndrome virus
EMA: The European Medicines Agency
BW: bodyweight
CLSI: The Clinical and Laboratory Standards Institute
VAST: Veterinary Antimicrobial Susceptibility Testing
EUCAST: The European Committee on Antimicrobial Susceptibility Testing
TSA: Tryptone soya agar
NAD: Beta-Nicotinamide adenine dinucleotide trihydrate
MIC_50_ and MIC_90_: MIC, inhibiting the growth of at least 50% and 90% of isolates in a test population
OD: Optical density
cfu: Colony forming unit
PTA: The probability of target attainment
